# A Case of Guillain-Barré Syndrome With Multiple Causative Factors in a Young Male

**DOI:** 10.7759/cureus.49745

**Published:** 2023-11-30

**Authors:** Ahmed Zaka, Ozra Dehkordi, Roger Weir, Mosunmola Oyawusi, Richard M Millis

**Affiliations:** 1 Neurology, Howard University Hospital, Washington D.C., USA; 2 Pathophysiology, American University of Antigua, St. Johns, ATG

**Keywords:** hepatitis, west nile virus, epstein-barr virus, cytomegalovirus, guillain-barré syndrome

## Abstract

Guillain-Barré syndrome (GBS), an immune-mediated disease of the peripheral nervous system, is mainly characterized by rapidly progressive ascending weakness of the limbs with reduced or absent deep tendon reflexes. The exact cause of GBS is unknown, but it often occurs after a gastrointestinal or respiratory infection. The present study represents a case of GBS in which multiple antecedent antigenic stimuli may have contributed to the development of GBS.

The patient, a 28-year-old immunocompetent man with no significant medical history, presented to the emergency department (ED) with acute ascending flaccid paralysis that persisted for a few days. His initial symptoms included tingling in his legs, which started at his shin and calf and developed into numbness, which extended to his upper limbs and arms. A CT scan of the lumbar and cervical spine indicated minor L4-L5 and L5-S1 disc herniation as well as slight bulging in C5-C6 and C7. The patient was discharged but returned to the ED for urgent treatment the next day after he weakened rapidly, losing the ability to walk or maintain balance. Based on his clinical presentation of ascending weakness and generalized hyporeflexia, he was diagnosed with GBS. Abnormal liver function and positive blood tests for anti-cytomegalovirus (anti-CMV) and anti-Epstein-Barr virus (anti-EBV) IgG and IgM antibodies diagnosed hepatitis, CMV, and EBV, respectively. The patient was treated with intravenous immunoglobulin therapy (IVIG; 27 g/day) and antiviral medicine (ganciclovir; 340 mg IV/day) for five days. His nonexistent deep tendon reflexes began to improve two to three days following treatment. He was able to ambulate longer distances with a walker, and his upper extremities regained full strength. This case highlights the importance of a multiple-treatment approach to the treatment of GBS, wherein multiple antigenic triggering factors may be involved.

## Introduction

Guillain-Barré syndrome (GBS), an acute immune-mediated neuropathy, is an autoimmune disease of the peripheral nervous system triggered by an antecedent infection, surgery, injury, or reaction to immunization or inflammatory process [1,2]. The antibodies and inflammatory cells produced cross-react with epitopes on peripheral nerves and roots, leading to demyelination and/or axonal damage [3]. Different varieties of GBS have been identified in the literature based on unusual patient presentations and underlying infections. The infections known to trigger the syndrome include bacteria such as *Campylobacter jejuni* and *Haemophilus influenzae*, as well as viruses such as cytomegalovirus (CMV), Epstein-Barr virus (EBV), hepatitis E virus, Zika virus, SARS-CoV-2, and COVID-19 vaccination [4-9]. The present case report is unique in that it represents a case of GBS involving multiple antecedent infections.

## Case presentation

**Figure 1:**
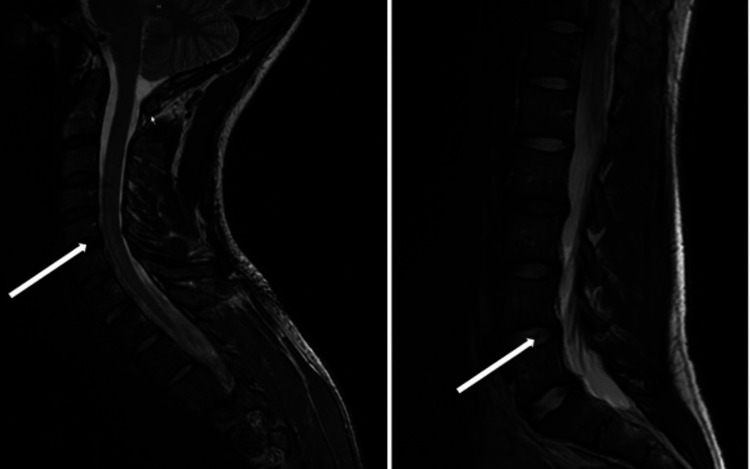
MRI of the cervical and lumbar spines indicated C5-C6, C7 slight bulging, and minor L4-L5 and L5-S1 disc herniation.

A 28-year-old man with no significant medical history was in normal health until a few days prior to the onset of symptoms when he had a strenuous workout at the gym and ate what he believed to be spoiled meat and spinach. The patient had an upper respiratory infection two weeks earlier and recently received a COVID-19 booster vaccine. He began to feel tingling in his shin and calf muscles, which developed into numbness and extended to his upper limbs and arms, causing him to go to the ED. He underwent a CT scan and later an MRI scan of the cervical and lumbar spine, which indicated minor L4-L5 and L5-S1 disc herniation and bulging, as well as C5-C6 and C7 slight bulging (Figure 1) but no evidence of spinal cord compression.

The patient was discharged but continued to suffer numbness in his legs and felt as if he was losing control of his legs throughout the day, forcing him to return to the ED the next day for urgent treatment. On his second visit, the patient's shin and calf continued to be numb and tingly. He couldn't keep his equilibrium and fell once. Additionally, he started having shallow breathing and shortness of breath. Other associated symptoms included intermittent dysphagia, tongue numbness, palpitations, and intermittent slurring of words. The patient denied having fever, chills, nausea or vomiting, chest pain, or changes in bowel and urinary habits. He had no recent travel and denied tick bites or urinary or bowel incontinence. He was admitted to the neurology intensive care unit with the tentative diagnosis of ascending polyneuropathy (GBS).

Physical examination

At the time of admission, the patient’s initial vital signs revealed a blood pressure of 145/86 mmHg, a heart rate of 88 beats per minute, a respiratory rate of 18 breaths per minute, a temperature of 97.8 °F, and an oxygen saturation of 98%. The cardio-pulmonary assessment was unremarkable. The throat examination did not reveal any erythematous changes or edema.

Neurological examination

**Figure 2:**
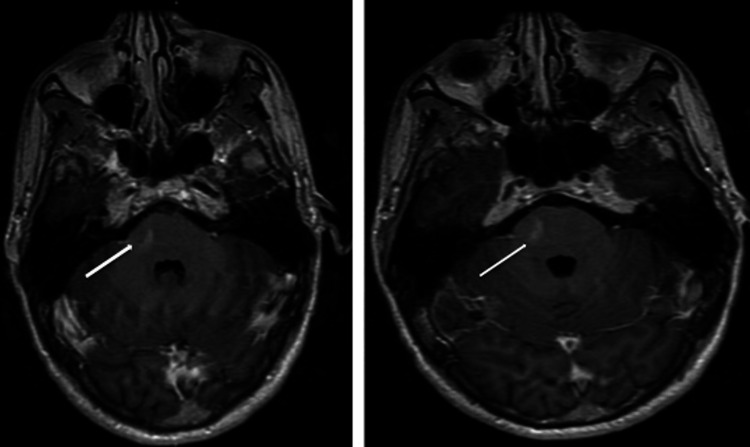
Post-contrast axial T1 MRI images show a focal 1.0 x 0.4 cm area of enhancement in two consecutive sections in the right pons of uncertain etiology.

On neurological examination, the patient was awake, alert, and oriented. His understanding of language was excellent. Cranial nerves were intact. His pupils were uniform, spherical, and light-sensitive. The visual fields were intact. Muscle mass and tone were also normal. The muscle strength in his upper and lower extremities was 4/5. He had no dysarthria. Deep tendon reflexes were absent in both lower extremities. He did not exhibit finger-nose-finger or heel-knee-shin dysmetria. Rapid, alternating movements were normal. His gait was unstable, and he was unable to keep his balance. The sensations on his upper extremities were intact, but touch and pinprick sensations were reduced on his lower extremities. A head CT showed no acute intracranial pathology. A brain MRI showed no evidence of acute territorial infarction, intracranial hemorrhage, or mass. A focal 1.0 x 0.4 cm area of enhancement in the right aspect of the pons of uncertain etiology was detected (Figure 2).

Multiple other images in a full MRI of the brain did not show this abnormality, and the patient was referred for a follow-up MRI.

Blood work

The white blood cell count was 6.93 x 10*3 u/L (range: 4.5-11.0 x 10*3 u/L). Liver function tests showed alanine transaminase of 111 IU/L (range: 0-55 IU/L), aspartate transaminase of 66 IU/L (range: 0-50), and creatine phosphokinase of 259 IU/L (range: 35-230 IU/L). Bilirubin and serum protein levels were normal. The serologic workup was negative for HIV, hepatitis A and C, and Lyme disease but positive for hepatitis B, EBV, and CMV. Serologic studies for CMV revealed IgM levels of >240 AU/mL (range: <0.30 AU/mL) and IgG levels of 3.30 U/mL (range: <0.60). Serological studies for EBV showed IgM levels > 160 U/mL (range: <36 U/mL) and IgG levels > 750 U/mL (range: <18 U/mL). Cerebrospinal fluid (CSF) was negative for myelin protein, <2.0 mcg/L (range: 2.0-4.0 mcg/L). The CSF total protein was 252.9 mg/dl (range: 15-45 mg/dl). The anti-West Nile virus IgG antibody was 2.99 U/ML (range: <1.30 U/ML), but the anti-West Nile virus IgM antibody was undetected. The patient also had Lyme disease IgG reactive for one type of antibody (Lyme 41KD AB+). On the basis of CSF total protein, serologic studies of CMV and EBV infection, and neurological manifestations, a diagnosis of CMV-EBV-induced GBS was confirmed. The patient was treated with intravenous immunoglobulin therapy (IVIG; 27 g/day) and the antiviral medicine ganciclovir (340mg IV/day) for five days. The patient was also given Solu-Medrol (60mg) for three days to help with the malaise and pain associated with the IVIG and gabapentin (300mg) for neuropathic pain. The patient had Lyme disease IgG reactive for one type of antibody (Lyme 41KD AB+). Although he did not meet the criteria for Lyme disease, given his presentation with ascending quadriparesis and facial nerve palsy, the infectious disease doctor recommended treatment for Lyme disease (ceftriaxone 2 g IV daily). He stayed in the intensive care unit for the duration of the GBS therapy. His nonexistent deep tendon reflexes began to improve two to three days following the IVIG and antiviral medications, and his upper extremities regained full strength. The patient continued to have transaminitis with an unclear etiology. The differentials included viral hepatitis, including CMV hepatitis, HSV hepatitis, EBV hepatitis, autoimmune hepatitis, medication-related (IVIG) hemochromatosis, and Wilson disease. Gastroenterology was consulted, who recommended getting a liver biopsy to confirm the etiology of the hepatitis as the CMV polymerase chain reaction (PCR) test came back negative. The patient refused a liver biopsy. He continued getting physical therapy in the hospital and was able to ambulate longer distances around the room with the walker.

## Discussion

The most common infectious diseases known to trigger GBS are *Campylobacter jejuni*, CMV, EBV, hepatitis E virus, and *Mycoplasma pneumoniae* [4-7,10-13]. Recent studies have also reported GBS as one of the complications of SARS-CoV-2 infection and COVID-19 vaccination [8,9]. The present report is unique in that it describes a case of GBS in an immunocompetent patient in which multiple antecedent antigenic factors may have contributed to the development of GBS. The CMV and EBV were diagnosed on the basis of positive serum anti-CMV IgG and IgM antibodies. The anti-Lyme disease IgG was reactive for one type of antibody (Lyme 41KD AB+), and there were also high levels of anti-West Nile virus IgG antibody in the CSF. In addition, the patient reported that he received a COVID-19 vaccine booster prior to his GBS.

Thus, in the treatment of this case, we initiated multiple treatment strategies, including intravenous immunoglobulin, antiviral, and antibacterial therapies. Two to three days following the administration of the IVIG and antiviral medication, the patient’s deep tendon reflexes began to improve, his upper extremities returned to full strength, and he was able to ambulate with a walker. On discharge, the patient was scheduled to get outpatient electromyographic and nerve conduction studies and was asked to follow up with the physical medicine and rehabilitation department.

## Conclusions

This report represents the case of a healthy, immunocompetent young adult man in whom multiple antecedent antigenic factors may have contributed to the development of GBS. The combination of IVIG, antiviral, and antibacterial medication improved the clinical outcome, suggesting that a multiple-treatment approach may be effective in the treatment of GBS where multiple antigenic triggering factors may be involved.
